# Diabetic Peripheral Microvascular Complications: Relationship to Cognitive Function

**DOI:** 10.1155/2011/723434

**Published:** 2011-11-17

**Authors:** Lorraine Ba-Tin, Paul Strike, Naji Tabet

**Affiliations:** ^1^Institute of Postgraduate Medicine, Brighton and Sussex Medical School, Falmer, Brighton BN1 9PH, UK; ^2^Research Design Service, South West, Royal Devon & Exeter Hospital, Exeter EX2 5DW, Devon, UK; ^3^Cognitive Treatment & Research Unit, Sussex Partnership NHS Foundation Trust, Crowborough, E. Sussex TN6 1NY, UK

## Abstract

Peripheral microvascular complications in diabetes are associated with concurrent cerebrovascular disease. As detailed cognitive assessment is not routinely carried out among diabetic patients, the aim was to establish whether the presence of clinical “peripheral” microvascular disease can identify a subgroup of patients with early evidence of cognitive impairment. Detailed psychometric assessment was performed in 23 diabetic patients with no microvascular complications (Group D), 27 diabetic patients with at least one microvascular complication: retinopathy, neuropathy, and/or nephropathy (Group DC), and 25 healthy controls (Group H). Groups D and DC participants had significantly lower scores on reaction time (*P* = 0.003 and 0.0001, resp.) compared to controls. Similarly, groups D and DC participants had significantly lower scores on rapid processing of visual information (*P* = 0.034 and 0.001, resp.) compared to controls. In contrast, there was no significant difference between Groups D and DC on any of the cognitive areas examined. The results show that diabetes, in general, is associated with cognitive dysfunction, but the additional presence of peripheral microvascular disease does not add to cognitive decline. The study, however, indirectly supports the notion that the aetiology of cognitive impairment in diabetes may not be restricted to vascular pathology.

## 1. Background

Diabetes mellitus is a common and serious disorder. The prevalence of the disease is projected to continue to increase significantly worldwide over the next few decades [1]. This lifelong endocrine disorder of relative or absolute insulin deficiency predisposes the diabetic person to many complications most of which are chronic in nature and some are life threatening. In particular, diabetes increases the risk of microvessel disease [2, 3]. As a result, serious conditions such as retinopathy, neuropathy, and nephropathy are not infrequently encountered among patients with diabetes.

In recent years, interest has also been directed towards another potential complication of diabetes, namely, cognitive decline. Increasing epidemiological evidence has linked diabetes with cognitive decline and dementia [4–11]. It has been suspected that the detrimental effect of diabetes on cognition is mediated through cerebrovascular disease. Recent evidence, for example, has shown that the brain of dementia patients with diabetes had more microvascular infarcts compared to the brain of dementia patients without diabetes [12]. However, diabetes is also now identified as a risk factor for both Alzheimer's disease (AD) and vascular dementia [13]. 

Many of the clinical complications of diabetes are caused by small and large vessel pathology [14]. In particular, “peripheral” microvascular complications of diabetes arising outside the brain appear to be correlated with corresponding microvascular changes in the brain. For example, diabetic retinopathy and retinal microvascular abnormalities were associated with various MRI signs such as small focal white matter hyperintensities and lesions [15–18]. Likewise, the presence of microalbuminuria in the general population has been associated with significantly lower cognitive function score [19]. 

The association between diabetes and cognitive impairment is important not just from an aetiological perspective, but also from the standpoint of day to day practical management of the disorder. The presence of dementia is likely to have a significant impact on the self-care and independency of patients [20]. It will also have significant influence on important aspects of treatment including the administration of medication (tablets and insulin) and blood sugar monitoring [21]. Investigations and subsequent clinical diagnosis of microvascular complications such as nephropathy, retinopathy, and neuropathy are regularly made in patients with diabetes. However, early investigations of potential cognitive impairment at specialist memory clinics are not routinely undertaken in patients who do not show obvious signs of dementia. The early identification of clinically relevant cognitive impairment in diabetic patients is essential because of available symptomatic treatment, the need to educate patients and carers, and the need to instate required supportive measures.

Microvascular complications in diabetes arising outside the brain are associated with concurrent brain pathology. However, it remains unclear whether this translates into early evidence of cognitive abnormalities beyond what is observed in diabetic patients free from such complications. Hence, the main aim of this study was to assess whether the presence of “peripheral” microvascular complications in patients with diabetes pointed towards evidence of early cognitive decline. Such data is important to assess whether the clinical diagnosis of microvascular complications in organ systems other than the brain identifies a subgroup of diabetic patients showing early sign of cognitive failure. In addition, such information may also contribute to increasing our understanding on the role of microvessel disease in the genesis of cognitive dysfunction in diabetes.

## 2. Methods

### 2.1. Participants

Seventy-five participants were recruited into three study groups: 25 healthy nondiabetic controls (Group H), 27 diabetic patients without the clinical presence of microvascular disease complications (Group D), and 23 diabetic patients with at least one peripheral microvascular complication such as nephropathy, retinopathy, and/or neuropathy (Group DC). 

Group H participants were recruited from diabetic case-group relatives or those who were attending community health clinics but who met all the inclusion and exclusion criteria and did not have diabetes. Groups D and DC participants were recruited from a mixture of community and hospital clinics. All diabetic participants in groups D and DC had a documented diagnosis of diabetes and were under the care of hospital diabetes service. The diagnosis of microvascular complications was obtained and classified using clinical, blood, and imaging investigations. In addition to a diagnosis of diabetes, group DC had a documented diagnosis of at least one of the following microvascular disease complications: retinopathy, nephropathy, and/or peripheral neuropathy. Such a diagnosis was established prior to the commencement of this study and was made or confirmed by experts. Hence, the diagnosis of retinopathy was made by ophthalmologists and the diagnosis of nephropathy was made by nephrologists. The diagnosis of peripheral neuropathy was confirmed by peripheral limb being insensate to 10 g microfilament, which is the gold standard assessment tool currently. Those who had uncertain diagnosis of vascular complications or were still under investigations to confirm such a diagnosis were not recruited to this study. Likewise, those with suspected preclinical microvascular disease but with no clear clinical manifestations were also excluded. Participants who had evidence of rheumatoid arthritis, cerebrovascular accidents, myocardial infarction, peripheral arterial disease, alcohol dependency, and depression were excluded from the study. None of the participants had a diagnosis of dementia in any of its stages or subtypes. Approval for this study was sought and obtained from the local Ethics and Research and Development committees. 

### 2.2. Cognitive Testing

Cognitive testing was carried out by using a touch-screen-computerised battery of psychometric tests. The Cambridge Neuropsychological Test Automated Batteries (CANTAB) [22] was used as a validated battery of computerised tests assessing a comprehensive range of cognitive functions such as processing speed, memory, learning, spatial, and digital reasoning. CANTAB has the advantage of not being culturally or language sensitive and tests scores are standardised from a store of normative data. It also has the advantage of having been used widely in other research fields and tested for reliability and validity. The CANTAB battery of tests included assessment of several cognitive domains: motor skills (MOTs), pattern recognition memory (PRM), spatial recognition memory (SRM), paired associate learning (PAL), reaction time (RT), match to sample visual search (MTS), and rapid visual information processing (RVP). Such testing is more likely to detect subtle cognitive deficits compared to other more widely used screening tests in clinical settings. 

Immediately before testing, participants were interviewed for current demographic data and assessed for the clinical diagnosis of a small vessel disease. Patients were included in either the D or DC groups solely based on the presence or absence of clear clinical diagnosis of microvascular complications. Data was recorded on the data collection sheet annotated with the confidential project number. 

### 2.3. Statistics

Quantitative data was collected from each participant and from each CANTAB test. The data exhibited clearly nonnormal distributions (predominantly a left-tail skew) in consequence of which nonparametric test procedures were used throughout. The Kruskal-Wallace analysis of variance by ranks was used initially to test for between-group differences in cognitive function [23]. An apparent age difference between the groups (see Table 1) led to repeat the between-group tests using analysis of covariance (ANCOVA) to adjust for any possible confounding by age. In keeping with the use of nonparametric methods throughout, the ANCOVA was run using rank transformed test data, following an earlier described procedure [24]. All analyses were undertaken using the software package Minitab v.14, and all tests were made at a 5% significance level (*P* < 0.05). As this was an essentially exploratory study, no adjustment to the significance level was made to control for multiple testing.

The sample size was estimated using the industry standard sample size calculation software package nQuery Advisor Release 4.0. The nonparametric size module was used for independent group comparisons (Mann-Whitney and Kruskal-Wallis test procedures) assuming a two-sided test significance level of 5%, and a specified test power of 80%. A difference between group-median values of at least 10% of scale range was proposed as the effect of interest to be detected, should such a difference exist. 

## 3. Results

Seventy-five individuals took part in the study and were subsequently divided into 3 groups. Demographic characteristics of participants are presented in Table 1. Noticeably, the mean age of the control group was significantly lower than that of diabetic groups (*P* = 0.026). Hence, any differences observed in cognitive scores among groups could have been a consequence of the disparity in age, that is, age may be a confounding factor. As a result, the study data was subsequently reanalyzed using an ANCOVA with age entered as covariate. Both types 1 and 2 diabetes patients were recruited to this study. Whilst Group D included mostly type 2 diabetics, Group DC included mostly those who had type 1 diabetes. Group D and DC did not differ significantly on the routinely measured HbA1c (mean 7.73 and 7.53, resp.; *P* = 0.97) and on the body mass index BMI (mean 30 and 32, resp.; *P* = 0.40). 

The results obtained on the 7 different CANTAB test domains are presented in Table 2. Significant differences between the groups were obtained in the domains of PRM, RT-5 reaction time and RVP. Following adjustment for possible confounding by age using nonparametric ANCOVA, significant differences between the control, and diabetes groups remained for RT and RVP but not for PRM: *P* = 0.102 (initially 0.024). 

Group H differed significantly from both diabetic groups on two of CANTAB domains, namely, RT and RVP. Interestingly, there was no significant difference between Group D and Group DC on these two or any other CANTAB domains (Table 3).

## 4. Discussion

Diabetes is a common disorder with a range of serious and potentially life-threatening complications. Many of these complications are clearly mediated through the disease's toxic effects on blood vessels. To date, however, the neuropathological mechanisms which may contribute to the observed cognitive impairment in diabetes remain unclear. 

There is growing body of evidence linking non-brain microvascular complications with cerebrovascular disease findings in patients with diabetes [15–18]. It was therefore of great interest to establish whether the presence of clinical “peripheral” microvascular disease identified a subgroup of diabetic patients with worse cognitive scores and potentially in need of early investigations, interventions, and help. The results of this study show that the additional presence of clinically evident microvascular disease complications did not significantly affect cognitive scores. This is an important finding which was not expected and which demands future study and assessment. It has been reported that the presence of white matter lesions is associated with cognitive decline [25]. However, the true impact of such lesions on the development of dementia is far from being clearly established. Recently, for example, no significant association was found between cerebrovascular disease severity as reported on MRI scans and cognitive scores in AD patients [26]. 

The results obtained in this study have shown that patients with a diagnosis of diabetes, but who do not suffer from a dementia illness, have some early evidence of cognitive deficits. Patients with diabetes, with and without microvascular complications, performed significantly worse on reaction times and on rapid visual processing which require focussed attention for a prolonged period. These results support the notion that diabetes negatively affects some aspects of cognition, a process which may lead in some to dementia. These results add weight to the various epidemiological and retrospective studies which have shown a link between diabetes in general and dementia.

MRIs of the brain are not routinely ordered as part of assessment of diabetes. In fact, diabetic patients are not routinely evaluated for cognitive outcome [27]. Undoubtedly, the presence of cognitive impairment in diabetes may have significant impact on the day to day management of these patients; not least in the area of insulin and drug administration. However, the findings presented here indicate that the clinical diagnosis of “peripheral” microvascular disease cannot be used to identify patients with early cognitive impairment beyond what is seen among all diabetic patients. It is still not known, however, whether such patients are more likely to develop dementia at some time in the future. It is hoped that such information will become available in the near future from a current long-term study [28]. In this study, no significant difference was noted in any of the seven cognitive domains studied between the DC and D Groups despite the fact that patients in the DC group had diabetes for longer duration compared to the D group. 

As no significant difference in cognitive scores existed among diabetes patients with and without microvascular complications, it is tempting to speculate about the role of microvascular pathology in the development of dementia among diabetic patients. A recent study has shown that medial temporal lobe atrophy was associated with diabetes independently of the amount of small vessel disease [29].While the direct toxic effect of hyperglycaemia on blood vessel pathology cannot be doubted, other mechanisms may also contribute to brain damage in diabetes leading to cognitive impairment. These may include the formation of advanced glycation end products [30], inflammation [31], insulin-induced amyloid pathology [32, 33], and neurofibrillary tangle formation [34]. 

This study has several limitations which need to be considered. The relatively small number of participants in each of the 3 study groups did not allow for meaningful subgroup evaluation in relation to diabetes types 1 and 2. In this study, diabetes patients had both types 1 and 2, as has been the case in many other studies [35]. However, the prevalence of type 1 was higher in the DC group. Both types of diabetes are associated with poor scores on cognitive testing. In type 1 there is decrease in mental speed and mental flexibility [36], while in type 2 diabetes in addition to mental speed and mental flexibility there may be deficits in learning and memory [37, 38]. Autopsy data based on the Honolulu-Asia Aging Study cohort shows a significant association of type 2 diabetes with hippocampal neurofibrillary tangles and neuritic plaques [39]. Korf et al. [9] found that in addition to increased vascular brain damage, type 2 diabetes also caused hippocampal atrophy. In future studies, large number of participants will be needed to clearly establish a difference between the two types of diabetes. The study design also lacked prespecified matching criteria for the control group. As a result, in this case-control study unintentional bias sampling may have resulted in observations by chance. These limitations need to be considered when interpreting the results. Therefore, in addition to a larger number of participants in each subgroup, future research would benefit from stricter sampling design which may limit any issue of bias. This should include a similar duration of diabetes among the groups which is not the case for this study. 

Both hypertension and diabetes are known risk factors for vascular disease [10]. As both conditions are more prevalent among the old, they are also more likely to coexist in the same patient. In this study, hypertension was present in both diabetes groups but was more prevalent in the DC group compared to the D group (22 and 7 retrospectively). The increase prevalence of hypertension in the DC group does not appear to have added significantly to worsening cognitive scores. More research is still needed to understand further the potential interaction between diabetes and hypertension in the development of dementia. The same applies for dyslipidemia, which may be a complicating factor. Its relationship to microvessel disease in the presence of diabetes needs to be understood further. Another limitation which also needs to be recognised is that pathological processes leading to microvascular complications in diabetes may be present for a significant time before clinical manifestations become evident. In the current study, all patients in the DC group had an established clinical diagnosis of microvascular disease. Hence, the results obtained are specific to clinical microvascular disease only. The presence of carotid stenosis may significantly influence the state of micro- and macrovascular disease of the brain. In the current study, none of the participants had symptomatic carotid stenosis. However, asymptomatic carotid stenosis may represent a confounding factor. The state of the carotid arteries was not assessed specifically for this study. Future work would need to incorporate ultrasound examination of these arteries. 

There has been interest in recent years in establishing whether the degree of glycemic control may influence cognitive function. Cukierman-Yaffe et al. (2009) [40] reported a statistically significant correlation between measured HbA1c levels and scores on four cognitive assessments. This initial report highlights the need for stricter glycemic control and its benefit to cognition. In this study HbA1c did not differ significantly between the two diabetes groups. As a result, our findings are unlikely to have been influenced by the degree of glycemic control. However, previous data, confirmed indirectly by this study, support a pathological role for hyperglycemia in the pathogenesis of cognitive dysfunction independent of cerebrovascular disease. The importance of glycemic control needs to be emphasised.

The main aim of this study was to assess whether the presence of microvascular complications arising outside the brain proper identifies a subgroup of diabetic patients with a worse cognitive profile which would not necessarily be detected by the widely used brief cognitive screening tests [41, 42]. Such information is clinically important to help in the management of such patients. It was not the aim to directly assess cerebrovascular disease in diabetes. Many studies addressing this issue have been published previously. 

Notwithstanding some of the limitations in the current study, the data presented here are important in two aspects. Firstly, clinically relevant microvascular complications arising outside the brain are not associated with a poorer cognitive function among diabetic patients. Hence, based on these results, which will need to be confirmed by other studies, no additional cognitive investigations are warranted in such patients solely based on the presence of such microvascular complications; that is, when no cognitive impairment is clinically suspected. Secondly and indirectly, the results add weight to the notion that mechanisms not restricted to microvascular pathology may be responsible for the associated cognitive impairment observed in diabetes. 

## Figures and Tables

**Table 1:** Demographic data and social characteristics of participants.

Characteristics	Healthy group (H) (*n* = 25)	Diabetics without complications (D) (*n* = 23)	Diabetics with complications (DC) (*n* = 27)
	Range	Range	Range
Mean age (years)	53 (41–68)	60 (45–80)	61 (42–80)
Male : Female	11 : 14	14 : 11	14 : 11
Ethnicity (non-Caucasian)	0	0	0
School years	11	10.6	10.9
Smokers	0	0	0
Diabetes type 1 : 2	0	6 : 19	17 : 9
Diagnosed diabetic (years)	0	7.4 (1–27 years)	20.8 (1–46 years)

**Table 2:** CANTAB test data for the three study groups across the various test domains. Pattern recognition memory (PRM), reaction time (RT), and rapid visual information processing (RVP) differed among the study groups with only RT and RVP remaining significantly different after adjusting for age.

Test	Group	min	25th centile	Median	75th centile	max	*P* value Kruskal-Wallis ANOVA	95% CI (median)
PRM	H	−2.048	−0.338	0.495	0.919	1.286		−0.179 to +0.581
	D	−2.5	−1.452	−0.282	0.495	1.023	0.024	−1.300 to +0.319
	DC	−3.1	−0.879	−0.338	0.495	1.343		−0.752 to +0.441

SRM	H	−4.136	−0.639	0.0278	0.317	1.923		−0.560 to +0.275
	D	−2.747	−1.139	−0.140	0.360	1.878	0.86	−1.062 to +0.360
	DC	−4.136	−1.360	−0.275	0.360	1.415		−0.897 to +0.344

PAL	H	−1.652	−0.492	0.214	0.530	1.271		−0.292 to +0.447
	D	10.419	−2.928	0.0976	0.695	1.017	0.63	−0.250 to +0.695
	DC	11.182	−2.831	0.000	0.508	1.017		−1.187 to +0.390

MTS	H	−3.808	−1.351	−0.307	0.686	1.170		−1.027 to +0.431
	D	−6.271	−0.997	0.322	0.521	1.176	0.89	−0.903 to +0.467
	DC	−4.001	−1.046	−0.247	0.940	1.170		−0.841 to +0.505

RTmove	H	−2.890	−0.003	0.466	1.060	1.435		+0.094 to +0.965
	D	−2.407	−0.641	0.217	1.025	1.573	0.43	−0.639 to +0.812
	DC	−331.3	−0.572	0.434	1.010	1.970		−0.330 to +0.838

RT reaction time	H	−1.476	−0.148	0.515	0.797	1.847		+0.076 to +0.766
	D	−9.092	−1.150	−0.354	0.248	1.578	0.003	−1.307 to −0.018
	DC	−4.299	−1.496	−0.525	−0.156	1.199		−1.462 to −0.210

RVP	H	−1.773	−0.846	0.025	0.689	1.595		−0.702 to −0.577
	D	−1.904	−1.713	−1.037	0.217	1.221	0.001	−1.698 to −0.151
	DC	−2.644	−1.621	−1.007	−0.344	1.782		−1.538 to −0.410

**Table 3:** Analysis of group differences for RT and RVP. Group H differed significantly from Groups D and DC on both measures. However, there was no significant difference between Group D and Group DC on either of the cognitive tests.

RT	
Mann-Whitney	Significance
H versus D	*P* = 0.003
H versus DC	*P* = 0.0001
D versus DC	*P* = 0.624

RVP

Mann-Whitney	Significance
H versus D	*P* = 0.034
H versus DC	*P* = 0.001
D versus DC	*P* = 0.576
